# 

**Published:** 1869-05-15

**Authors:** 


### *CONTENTS.*

REVIEWS:

Monthly Review of Medical and General Science in France and Europe... 302
General Science... 310
Editorial Notes... 312

ORIGINAL COMMUNICATIONS:

Autopsy... 321
Three Cases of Trichnasis in Clay County, Ill... 322
Super-Oxygenation... 324

EDITORIAL:

Prize of the Alumni Association of the Chicago Medical College... 325
Board of Health... 326
Cranitomy vs. Forceps... 327

PROCEEDINGS OF SOCIETIES:

Minnesota Siate Medical Society... 327
DeWitt County Medical Society... 329

LOOT:

Chestnut Leaves in Hooping Cough... 330
Obscure Cases of Sudden Death... 330

